# Prevalence of Autonomic Dysfunction and Correlation with Markers of Disease Severity in Cardiac Amyloidosis

**DOI:** 10.3390/jcm14134682

**Published:** 2025-07-02

**Authors:** Michael Poledniczek, Konstantin Hölzl, Christina Kronberger, Nikita Ermolaev, Lena Marie Schmid, René Rettl, Christina Binder, Luciana Camuz Ligios, Mahshid Eslami, Christian Hengstenberg, Roza Badr Eslam, Jutta Bergler-Klein, Johannes Kastner, Andreas Anselm Kammerlander, Franz Duca

**Affiliations:** 1 Department of Internal Medicine II, Division of Cardiology, Medical University of Vienna, 1090 Vienna, Austria; michael.poledniczek@meduniwien.ac.at (M.P.);; 2 Department of Neurology, Medical University of Vienna, 1090 Vienna, Austria; 3 Clinical Trials Coordination Centre, Medical University of Vienna, 1090 Vienna, Austria

**Keywords:** autonomic dysfunction, transthyretin amyloidosis, transthyretin amyloid cardiomyopathy

## Abstract

**Background**: Transthyretin amyloidosis is a multi-system disease that may manifest as cardiomyopathy (ATTR-CM) and/or polyneuropathy. Both disease manifestations are associated with autonomic dysfunction. However, the prevalence of autonomic dysfunction in ATTR-CM remains to be evaluated. **Methods**: Within the scope of a prospective ATTR-CM registry, the Composite Autonomic Symptom Score-31 (COMPASS-31) questionnaire was applied to consecutive patients between November 2022 and November 2024. Baseline characteristics are described, and associations of the COMPASS-31 score with markers of disease severity were assessed. Kaplan–Meier analysis was utilized to assess the COMPASS-31 score’s association with a combined endpoint of all-cause mortality and heart failure-related hospitalizations. **Results**: A total of 129 ATTR-CM patients [81.7 years (IQR: 77.4–84.3), 108 male (83.7%)] were included in the final study cohort. After stratification using the COMPASS-31 median [14 points, interquartile range (IQR): 6–29], statistically significant differences with regard to New York Heart Association (NYHA) stage and the Kansas City Cardiomyopathy Questionnaire (KCCQ) were observed. Furthermore, the COMPASS-31 score was moderately correlated with the KCCQ score in Spearman correlation analysis (r = −0.55, *p* < 0.001). The primary endpoint occurred in 16 patients (13 HF-hospitalizations/3 deaths) after 6.3 (IQR: 2.8–17.1) months. In Kaplan–Meier analysis, a COMPASS-31 score above the median of 14 was also associated with the primary endpoint of all-cause mortality and HF-related hospitalization (log-rank *p* = 0.047). **Conclusions**: Autonomic dysfunction is highly prevalent in ATTR-CM, affecting almost two-thirds of patients. As the presence of autonomic dysfunction is likely associated with more severely impaired quality of life, routine screening for this disease manifestation of transthyretin amyloidosis may be advisable.

## 1. Introduction

Cardiac amyloidosis (CA) is characterized by myocardial amyloid infiltration [1]. Subsequently, wall thickness increases, and myocardial contractility and relaxation become impaired, resulting in decreased cardiac output and heart failure (HF) [1]. The most important amyloid precursor proteins are light chains and transthyretin [1]. Having undergone steric conformation changes, precursor proteins aggregate and form amyloid light chain or amyloid transthyretin (ATTR), which is deposited in tissues and causes amyloidosis. Light chain amyloidosis evolves due to plasma cell dyscrasia, while transthyretin amyloidosis can be caused either by a pathogenic TTR gene variant (formerly hereditary TTR) or arise as an acquired disease of the elderly [1].

While cardiac involvement is a crucial determinant of outcome in amyloidosis [2,3], amyloidosis remains a systemic disease with manifestations beyond the heart. Patients with light chain amyloidosis will often develop renal involvement resulting in chronic kidney disease or kidney failure, gastrointestinal, vascular, or bone marrow involvement [4]. In contrast, ATTR amyloidosis primarily affects the myocardium and peripheral nervous system and may cause spinal canal stenosis or carpal tunnel syndrome due to infiltration of the ligamentum flavum or the carpal ligament [2,5]. In addition, gastrointestinal, skin, and ocular involvement are occasionally observed [5].

In addition to impaired physical capabilities, CA patients’ quality of life is impaired and associated with adverse outcomes [6,7]. While progressive peripheral sensorimotor polyneuropathy is well described in systemic amyloidosis [8,9], little is known about the prevalence and effects on autonomic dysfunction. We assume that autonomic dysfunction is a crucial determinant of quality of life and is currently not adequately represented in traditional quality of life questionnaires for HF patients. We therefore prospectively applied the Composite Autonomic Symptom Score-31 (COMPASS-31) to our ATTR cardiomyopathy (ATTR-CM) cohort.

## 2. Materials and Methods

### 2.1. Setting

The present analysis was performed at the Medical University of Vienna, Vienna, Austria, within the scope of a CA registry, which is approved by the institutional review board (#1079/2023) and implemented in compliance with the Declaration of Helsinki. All patients diagnosed with ATTR-CM according to the guidelines on cardiomyopathies by the European Society of Cardiology [10] were eligible for inclusion. Prior to inclusion, all patients provided written informed consent. Between November 2022 and November 2024, patients who presented to the institution’s dedicated CA outpatient clinic and participated in the registry were asked to complete the COMPASS-31 questionnaire and the Kansas Cardiomyopathy Questionnaire (KCCQ) as described below.

### 2.2. Quality of Life Questionnaires

The COMPASS-31 questionnaire is a non-disease-specific patient-reported outcomes measurement tool designed to assess signs and symptoms of autonomic dysfunction [11]. Originally developed as an 84-item version, it has been abbreviated to include only 31 questions in six domains, i.e., orthostatic intolerance, vasomotor dysfunction, secretomotor dysfunction, gastrointestinal, bladder, and pupillomotor dysfunction, from which a composite autonomic symptom score is calculated [11]. The abbreviated COMPASS-31 questionnaire was completed self-sufficiently in this study [11]. For the calculation of COMPASS-31 scores, where higher results indicate an increased symptom burden, the method proposed by Sletten et al. [11] was utilized. Currently, there are no established thresholds for autonomic dysfunction in ATTR-CM patients. For the purpose of this study, the screening threshold reported by Meling et al. [12] comprising 10 points was utilized.

The KCCQ is a quality-of-life measurement tool used for HF patients [13]. The KCCQ scale ranges from 100 (indicating ideal health-related quality of life) to 0 (worst imaginable quality of life) [13]. While it has been developed and extensively studied in general HF populations [14,15], we have previously also demonstrated its independent association with adverse outcome in transthyretin amyloid cardiomyopathy [6]. For this analysis, the abbreviated KCCQ-12 [16], comprising 12 items instead of 23 in the original KCCQ, was utilized.

### 2.3. Further Parameters of Interest

As part of routine baseline or follow-up in-clinic visits in our tertiary referral center, a complete hemogram, blood chemistry, and biomarkers of HF [N-terminal prohormone of brain natriuretic peptide (NT-proBNP), troponin T, and creatinine/estimated glomerular filtration rate (eGFR)] were compiled. All laboratory analyses were performed at the institution’s central laboratory (Department of Laboratory Medicine, Medical University of Vienna, Vienna, Austria), and the eGFR was estimated by applying the chronic kidney disease epidemiology collaboration formula [17]. Disease stage was assessed by applying the United Kingdom National Amyloidosis Centre (NAC) staging system’s criteria [18]. Medical history, comorbidities, and concurrent medication were compiled in the course of the in-clinic visits. Additionally, electronic health records were searched and analyzed if applicable. The presence of diabetes mellitus was assumed with evidence of a level of glycated hemoglobin (HbA1c) ≥ 6.5% without anti-diabetic medication [19] or with HbA1c < 6.5% in patients who receive anti-diabetic medication. The primary endpoint of the present analysis was a composite of (1) all-cause mortality and (2) HF-related hospitalization.

### 2.4. Statistics

Categorial variables are presented as numbers and percentages. Depending on the variables’ distribution, which was assessed using the Shapiro–Wilk test, continuous parameters are presented as mean and standard deviation (SD) or median and interquartile range (IQR). Cohort characteristics were compared between sub-cohorts stratified by the COMPASS-31 score median using the chi-square test and the t-test for independent samples or the Mann–Whitney U test as applicable. In secondary analysis, patients were stratified by sex and ATTRv status. Correlation between COMPASS-31 sub-scores and the KCCQ was assessed using Spearman correlation analysis. The association of parameters with the COMPASS-31 score was assessed using a step-wise linear regression model. Association of the COMPASS-31 score with the primary endpoint was tested by applying Kaplan–Meier analysis and calculation of the log-rank test as well as Cox proportional hazard regression analysis.

Statistical significance was defined as a confidence interval of 95% and a *p*-value of <0.05, respectively. Statistical analysis was conducted with using BlueSky Statistics 10.3.4, R package version 8.95 (BlueSky Statistics LLC, Chicago, IL, USA).

## 3. Results

**Figure 1:**
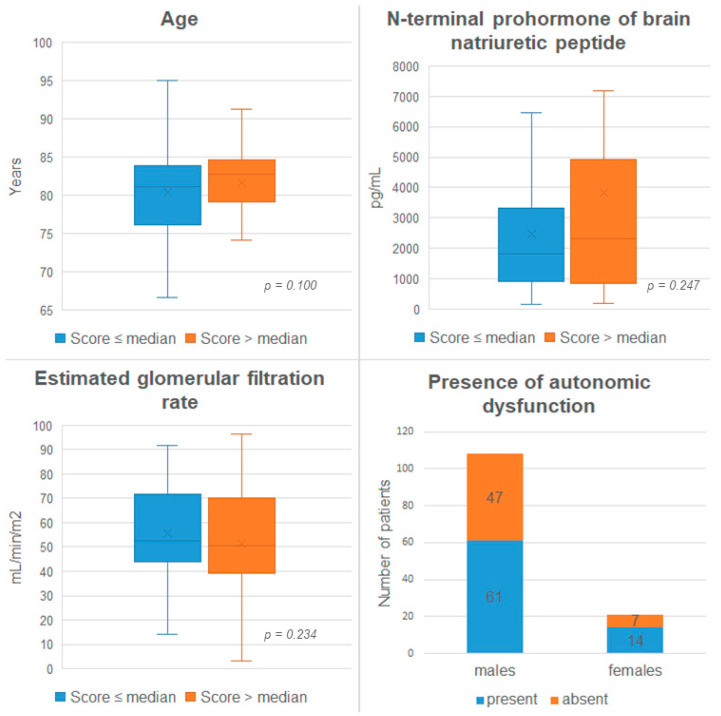
Baseline parameters stratified by the COMPASS-31 score median and the presence of autonomic dysfunction stratified by sex. X indicates the mean.

A total of 129 ATTR-CM patients [81.7 years (IQR: 77.4–84.3), 108 males (83.7%)] were included in the final study cohort. The median COMPASS-31 score was 14 (IQR: 6–29) and the full baseline characteristics are depicted in Table 1 and Figure 1. When applying the screening threshold of 10 points, 75 (58.1%) patients presented with COMPASS-31 results indicative of autonomic dysfunction.

When stratified by the COMPASS-31 score median, 62 patients constituted the patient cohort with more advanced autonomic dysfunction, while 67 patients scored a COMPASS-31 score equal to or less than the median of 14 [30 (IQR: 19–38) vs. 7 (IQR: 3–9), *p* < 0.001]. Statistically significant differences between these two groups were observed with regard to New York Heart Association (NYHA) functional class, where the patient group with a COMPASS-31 score > 14 reported more severe HF symptoms. In the cohort with higher COMPASS-31 score, a general tendency toward more severely elevated biomarkers of HF and higher loop diuretics doses was observed, and the comparisons failed to surpass the pre-defined level of statistical significance. Between the two cohorts, highly significant differences were observed with regard to all sub-categories of both the COMPASS-31 score as well as the KCCQ.

In our patient cohort, male individuals were less likely to exhibit a ATTR variant in genetic analysis [6 (5.6%) vs. 4 (19.0%), *p* = 0.036] and were more likely to be in less advanced disease stages assessed using the NAC staging system (*p* = 0.019). However, no significant differences with regard to COMPASS-31 or KCCQ (sub-)scores were observed. Compared to wild-type ATTR-CM patients, those with a pathogenic TTR gene variant were significantly younger [68.6 (IQR: (78.6–84.3) vs. 81.9 (64.5–78.5), *p* = 0.003] and more likely to be female (40.0% vs. 14.4%, *p* = 0.036), and no ATTR variant patients suffered from concomitant cardiac light chain amyloidosis (Table 2).

**Figure 2:**
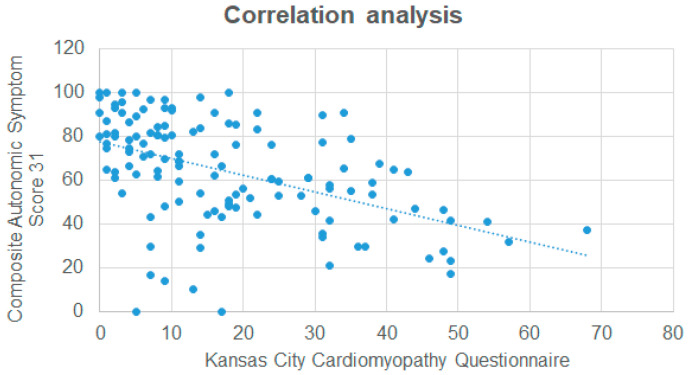
Correlation analysis between the Kansas City Cardiomyopathy Questionnaire and the Composite Autonomic Symptom Score 31.

In a Spearman correlation analysis (Table 3, Figure 2), significant associations were observed between all COMPASS-31 sub-scores and the KCCQ overall score. Interestingly, the associations were generally weak to moderate. In univariable linear regression analysis, only the NYHA stage and KCCQ score demonstrated a significant association with the COMPASS-31 score. In an adjusted multivariate model, only the KCCQ score remained significantly associated with the COMPASS-31 score. All results of the linear regression analysis are shown in Table 4.

**Figure 3:**
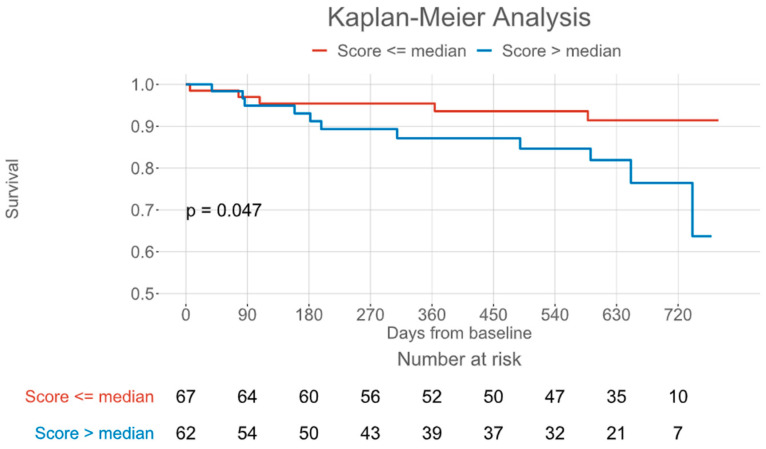
Kaplan–Meier plot for a composite of all-cause mortality and heart failure-related hospitalization, grouped by the Composite Autonomic Symptom Score 31 median.

The primary endpoint, a composite of all-cause mortality and HF-related hospitalizations, occurred in 16 patients (13 HF hospitalizations/3 deaths) after 6.3 (IQR: 2.8–17.1) months. The full observation period was 20.3 (IQR: 10.2–23.0) months. In Kaplan–Meier analysis, a COMPASS-31 score above the median of 14 was also associated with the primary endpoint of all-cause mortality and HF-related hospitalization (Figure 3, log-rank *p* = 0.047). In Cox regression analysis, the COMPASS-31 score failed to demonstrate significant association with the primary endpoint (*p* = 0.11).

## 4. Discussion

We demonstrated that autonomic dysfunction is highly prevalent in ATTR-CM patients. Relying on previously established thresholds [11], we estimate that almost two-thirds of ATTR-CM patients may be affected by autonomic dysfunction. This study adds to the existing literature as this is the first study to report COMPASS-31 results in wild-type ATTR-CM patients.

Our results suggest that autonomic dysfunction is not significantly associated with traditional assessments of disease severity, e.g., biomarkers of HF, or eGFR, and also not associated with age and sex. Rather, the only entity associated with autonomic dysfunction was quality of life assessed using the KCCQ. Therefore, we suggest that the degree of autonomic dysfunction is among the determinants of overall quality of life and may therefore deserve increased attention from treating physicians.

In clinical practice, symptoms of autonomic dysfunction might easily be overlooked. We speculate that this may be due to the relative difficulty of objectively assessing this aspect of the disease. Furthermore, autonomic dysfunction is diverse and varying in presentation and therefore hard to objectively assess utilizing a single measurement. Previous studies have approached this issue by utilizing heart rate variability in patients with cardiac light chain amyloidosis [20] or by measuring electrochemical skin conductance in ATTR-CM patients [21]. However, these assessments are likely to fail to assess autonomic dysfunction in its entirety. Beyond that, with a high prevalence of cardiac arrhythmia, especially atrial fibrillation [22], heart rate variability alone may not be suitable for assessing autonomic dysfunction in ATTR-CM patients.

While, depending on the genotype, neural involvement is generally a common feature in variant ATTR amyloidosis, in wild-type patients, it seems to appear less frequently, depending on the screening methods [23]. However, as amyloid deposition has been demonstrated to result in both the degeneration of neurons in the sensory and autonomic ganglia as well as atrophy of Schwann cells [24], some degree of neural involvement may be expected in wild-type ATTR patients as well. From findings in a small mixed cohort of patients with ATTR amyloidosis who underwent comprehensive neurological examination including skin biopsy, it can be concluded that 40% of wild-type patients demonstrated findings in line with large and/or small fiber neuropathy that could not be explained by comorbidities [25]. This also supports the notion that neurological involvement and concomitant symptoms are frequently underdiagnosed in these patients.

As a large proportion of ATTR-CM patients are of advanced age, comorbidities and their effect on autonomic dysfunction specifically, but also quality of life more broadly, need to be considered. Most importantly, poorly controlled diabetes mellitus may also lead to autonomic dysfunction and increased COMPASS-31 scores, as previously reported [26]. In our study, however, concomitant diabetes mellitus had no effect on the COMPASS-31 score. This suggests that cohort autonomic dysfunction was primarily due to ATTR amyloidosis in our study.

In a meta-analysis, Pearson and Smart [27] demonstrated that exercise rehabilitation may be suitable to improve heart rate variability, one aspect of autonomic dysfunction. This suggests that autonomic dysfunction is a modifiable aspect of disease and may be utilized as a potential endpoint in rehabilitation trials or quality-of-life focused intervention studies.

Finally, in line with previous studies [21], our results suggest that autonomic dysfunction is also linked to adverse outcome.

### 4.1. Limitations

As this analysis was conducted in a single tertiary referral center setting, certain biases with regard to patient selection cannot fully be excluded without confirmation of findings in larger, ideally multi-center cohorts.

The majority of participants in our analysis were wild-type patients and were only followed for a median of 20 months. A higher proportion of variant ATTR patients, longer follow-up, and repeated deployment of the COMPASS-31 would have likely increased the robustness of our analysis. As ATTR-CM patients are typically of advanced age, autonomic dysfunction could also be caused by concomitant disease or other unrecognized factors and should ideally be compared to a healthy control group or matched non-ATTR-CM HF patients.

Patient-reported outcome measurements are always susceptible to reporting bias and may fail to adequately represent patients who are not willing to answer questionnaires or fail to recall symptoms or estimate symptom frequency or severity. Finally, there are currently no reliable data on the added clinical benefit with the application of patient-reported outcome measurement tools in ATTR-CM.

### 4.2. Future Research

Currently, autonomic dysfunction is laborious to evaluate objectively. Objective examinations, e.g., skin conductance measurements, tilt-table testing, or Ewing’s battery, are unlikely to be easily established in clinical practice. Future studies ought to aim to validate tools that are routinely available in practice like the COMPASS-31 questionnaire or heart rate variability utilizing more objective measurements of autonomic dysfunction. In addition, longitudinal assessment of changes in the COMPASS-31 may help to better understand treatment effects, both of specific therapeutics or conventional concomitant therapy, e.g., loop diuretics. Furthermore, as autonomic dysfunction seems to be associated with subjective burden of disease, future randomized controlled trials should include the COMPASS-31 questionnaire to assess potential treatment effects on this aspect of ATTR-CM.

## 5. Conclusions

Autonomic dysfunction is highly prevalent in ATTR-CM and affects almost two-thirds of this patient population. As autonomic dysfunction is poorly correlated with other markers of disease severity including biomarkers of HF, routine screening for autonomic dysfunction may be considered, especially as the presence of autonomic dysfunction is likely associated with more severely impaired quality of life and worse outcomes.

## Figures and Tables

**Table 1:** Baseline characteristics of the cardiac amyloidosis cohort, stratified by the median COMPASS-31 score.

Variable	Total	COMPASS-31 Score > Median	COMPASS-31 Score ≤ Median	*p*-Value
	*n = 129*	*n = 62*	*n = 67*	
**Patient demographics**				
Age (years), median (IQR)	81.7 (77.4–84.3)	82.8 (79.3–84.6)	81.1 (76.1–83.6)	*0.100*
Female sex, *n* (%)	21 (16.3%)	13 (21.0%)	8 (11.9%)	*0.165*
ATTRv, *n* (%)	10 (7.8%)	5 (7.5%)	5 (8.2%)	*0.877*
AL, *n* (%)	3 (2.3%)	1 (1.6%)	2 (3.0%)	*0.627*
**Clinical parameters and laboratory evaluations**				
BMI, kg/m^2^, median (IQR)	25.3 (22.5–27.5)	23.9 (21.6–27.4)	25.8 (23.6–27.6)	*0.082*
NT-proBNP, pg/mL, median (IQR)	2091 (864–3695)	2325 (846–4866)	1820 (928–3327)	*0.234*
Troponin T, ng/L, median (IQR)	44.0 (30.0–70.0)	47.5 (32.5–73.2)	42.0 (27.0–58.5)	*0.139*
Creatinine, mg/dL, median (IQR)	1.2 (1.0–1.5)	1.2 (1.0–1.6)	1.2 (1.0–1.4)	*0.531*
eGFR, ml/min/1.73 m^2^, mean ± SD	53.6 ± 19.7	51.5 ± 21.5	55.5 ± 17.8	*0.247*
HbA1c, %, median (IQR)	5.8 (5.5–6.2)	5.8 (5.5–6.3)	5.7 (5.5–6.2)	
NYHA stage, *n* (%)				***0.001***
*1*	31 (24.0%)	6 (9.7%)	25 (37.3%)	
*2*	57 (44.2%)	30 (48.4%)	27 (40.3%)	
*3*	41 (31.8%)	26 (41.9%)	15 (22.4%)	
NAC stage, *n* (%)				*0.101*
*1*	64 (50.0%)	28 (45.2%)	36 (54.5%)	
*2*	36 (28.1%)	16 (25.8%)	20 (30.3%)	
*3*	28 (21.9%)	18 (29.0%)	10 (15.2%)	
**Medical history**				
Significant coronary artery disease, *n* (%)	27 (20.9%)	11 (17.7%)	16 (23.9%)	*0.392*
Atrial fibrillation, *n* (%)	85 (65.9%)	43 (69.4%)	42 (62.7%)	*0.425*
Atrial flutter, *n* (%)	8 (6.2%)	4 (6.5%)	4 (6.0%)	*0.910*
Diabetes mellitus, *n* (%)	27 (20.9%)	12 (19.4%)	15 (22.4%)	*0.672*
Arterial hypertension, *n* (%)	88 (68.2%)	44 (71.0%)	44 (65.7%)	*0.519*
**Medical therapy**				
Disease-modifying therapy, *n* (%)	88 (68.2%)	40 (64.5%)	48 (71.6%)	*0.385*
Disease-modifying therapy ≥ 6 months, *n* (%)	79 (61.2%)	36 (58.1%)	43 (64.2%)	*0.476*
SGLT2i, *n* (%)	58 (45.0%)	32 (51.6%)	26 (38.8%)	*0.228*
MRA, *n* (%)	57 (44.2%)	28 (45.2%)	29 (43.3%)	*0.830*
Loop diuretics, *n* (%)	78 (60.5%)	41 (66.1%)	37 (55.2%)	*0.206*
Loop diuretic dose, mg/kg, median (IQR)	0.24 (0.00–0.61)	0.30 (0.00–0.67)	0.19 (0.00–0.51)	*0.056*
**Questionnaire scores**				
**COMPASS-31 score, median (IQR)**	14 (6–29)	31 (19–38)	7 (3–9)	***<0.001***
*Score > cut-off, n (%)*	*75 (58.1%)*	*62 (100%)*	*13 (19.4%)*	***<0.001***
Orthostatic intolerance, median (IQR)	0 (0–16)	16 (9–23)	0 (0–0)	***<0.001***
*Any orthostatic intolerance, n (%)*	*55 (42.6%)*	*49 (79.0%)*	*6 (9.0%)*	***<0.001***
Vasomotor dysfunction, median (IQR)	0 (0–0)	0 (0–2)	0 (0–0)	***0.002***
*Any vasomotor dysfunction, n (%)*	*29 (22.5%)*	*21 (33.9%)*	*8 (11.9%)*	***0.003***
Secretomotor dysfunction, median (IQR)	2 (0–6)	6 (2–6)	0 (0–2)	***<0.001***
*Any secretomotor dysfunction, n (%)*	*77 (59.7%)*	*53 (85.5%)*	*24 (35.8%)*	***<0.001***
Gastrointestinal dysfunction, median (IQR)	4 (1–7)	6 (4–10)	2 (1–4)	***<0.001***
*Any gastrointestinal dysfunction, n (%)*	*113 (87.6%)*	*61 (98.4%)*	*52 (77.6%)*	***<0.001***
Bladder dysfunction, median (IQR)	1 (0–2)	2 (1–3)	0 (0–1)	***<0.001***
*Any bladder dysfunction, n (%)*	*79 (61.2%)*	*48 (77.4%)*	*31 (46.3%)*	***<0.001***
Pupillomotor dysfunction, median (IQR)	1 (0–2)	2 (1–2)	0 (0–1)	***<0.001***
*Any pupillomotor dysfunction, n (%)*	*87 (67.4%)*	*52 (83.9%)*	*35 (52.2%)*	***<0.001***
**KCCQ, median (IQR)**	66 (48–82)	54 (43–65)	80 (64–90)	***<0.001***
Physical limitations, median (IQR)	67 (50–83)	60 (39–81)	75 (58–83)	***0.004***
Symptom frequency, median (IQR)	75 (67–83)	58 (45–77)	85 (67–92)	***<0.001***
Quality of life, median (IQR)	75 (52–88)	50 (38–66)	75 (62–88)	***<0.001***
Social limitations, median (IQR)	67 (33–84)	50 (33–67)	75 (58–94)	***<0.001***

AL, (concomitant) light chain amyloidosis; ATTR, transthyretin amyloidosis; ATTRv, variant transthyretin amyloidosis; BMI, body mass index; COMPASS-31, Composite Autonomic Symptom Score-31; eGFR, estimated glomerular filtration rate; HbA1c, glycated hemoglobin; IQR, interquartile range; KCCQ, Kansas City Cardiomyopathy Questionnaire; MRA, mineralocorticoid receptor antagonist; NAC, National Amyloidosis Centre stage; NT-proBNP, N-terminal prohormone of brain natriuretic peptide; NYHA, New York Heart Association stage; SGLT2i, sodium-glucose cotransporter 2 inhibitor; SD, standard deviation. Categorical data are displayed as counts (percentage). Metric data are given as mean ± standard deviation in cases with a normal distribution and as median (interquartile range) in cases with a non-normal distribution, which is assessed utilizing the Shapiro–Wilk test. *p*-Values for metric variables are derived from the t-test for independent samples and the Mann–Whitney U test, respectively. For binary/categorical variables, the chi-square test is utilized.

**Table 2:** Baseline characteristics of the cardiac amyloidosis cohort, stratified by variant transthyretin gene status.

Variable	Total	ATTRwt	ATTRv	*p*-Value
	*n = 129*	*n = 118*	*n = 10*	
**Patient demographics**				
Age (years), median (IQR)	81.7 (77.4–84.3)	81.9 (78.6–84.3)	68.6 (64.5–78.5)	***0.003***
Female sex, *n* (%)	21 (16.3%)	17 (14.4%)	4 (40.0%)	***0.036***
AL, *n* (%)	3 (2.3%)	3 (2.5%)	0 (0.0%)	***<0.001***
**Clinical parameters and laboratory evaluations**				
BMI, kg/m^2^, median (IQR)	25.3 (22.5–27.5)	25.2 (22.8–27.5)	25.6 (20.2–28.8)	*0.947*
NT-proBNP, pg/mL, median (IQR)	2091 (864–3695)	2083 (920–3773)	2452 (884–3406)	*0.996*
Troponin T, ng/L, median (IQR)	44.0 (30.0–70.0)	43.0 (30.0–70.0)	54.0 (24.0–57.0)	*0.732*
Creatinine, mg/dL, median (IQR)	1.2 (1.0–1.5)	1.3 (1.0–1.5)	1.1 (0.8–1.7)	*0.236*
eGFR, ml/min/1.73 m^2^, mean ± SD	53.6 ± 19.7	52.7 ± 18.9	61.3 ± 26.8	*0.341*
HbA1c, %, median (IQR)	5.8 (5.5–6.2)	5.8 (5.5–6.2)	5.9 (5.6–6.3)	*0.399*
NYHA stage, *n* (%)				*0.592*
*1*	31 (24.0%)	30 (25.4%)	1 (10.0%)	
*2*	57 (44.2%)	50 (42.4%)	6 (60.0%)	
*3*	41 (31.8%)	38 (32.2%)	3 (30.0%)	
NAC stage, *n* (%)				*0.757*
*1*	64 (50.0%)	58 (49.6%)	5 (50.0%)	
*2*	36 (28.1%)	34 (29.1%)	2 (20.0%)	
*3*	28 (21.9%)	25 (21.4%)	3 (30.0%)	
**Questionnaire scores**				
**COMPASS-31 score, median (IQR)**	14 (6–29)	14 (6–27)	14 (6–35)	*0.606*
*Score > cut-off, n (%)*	*75 (58.1%)*	*69 (58.5%)*	*5 (50.0%)*	*0.602*
Orthostatic intolerance, median (IQR)	0 (0–16)	0 (0–16)	0 (0–16)	*0.870*
*Any orthostatic intolerance, n (%)*	*55 (42.6%)*	*50 (42.4%)*	*4 (40.0%)*	*0.884*
Vasomotor dysfunction, median (IQR)	0 (0–0)	0 (0–0)	0 (0–2)	*0.165*
*Any vasomotor dysfunction, n (%)*	*29 (22.5%)*	*24 (20.3%)*	*4 (40.0%)*	*0.149*
Secretomotor dysfunction, median (IQR)	2 (0–6)	2 (0–6)	2 (0–6)	*0.989*
*Any secretomotor dysfunction, n (%)*	*77 (59.7%)*	*71 (60.2%)*	*5 (50.0%)*	*0.530*
Gastrointestinal dysfunction, median (IQR)	4 (1–7)	4 (1–6)	6 (4–8)	*0.142*
*Any gastrointestinal dysfunction, n (%)*	*113 (87.6%)*	*103 (87.3%)*	*9 (90.0%)*	*0.803*
Bladder dysfunction, median (IQR)	1 (0–2)	1 (0–2)	1 (0–2)	*0.930*
*Any bladder dysfunction, n (%)*	*79 (61.2%)*	*73 (61.9%)*	*69 (60.0%)*	*0.907*
Pupillomotor dysfunction, median (IQR)	1 (0–2)	1 (0–2)	2 (0–2)	*0.313*
*Any pupillomotor dysfunction, n (%)*	*87 (67.4%)*	*79 (66.9%)*	*7 (70.0%)*	*0.844*
**KCCQ, median (IQR)**	66 (48–82)	65 (48–82)	77 (59–87)	*0.492*
Physical limitations, median (IQR)	67 (50–83)	67 (50–83)	75 (60–81)	*0.839*
Symptom frequency, median (IQR)	75 (67–83)	73 (52–88)	83 (59–89)	*0.687*
Quality of life, median (IQR)	75 (52–88)	62 (50–88)	75 (53–88)	*0.475*
Social limitations, median (IQR)	67 (33–84)	67 (33–83)	79 (52–92)	*0.472*

AL, (concomitant) light chain amyloidosis; ATTR, transthyretin amyloidosis; ATTRv, variant transthyretin amyloidosis; BMI, body mass index; COMPASS-31, Composite Autonomic Symptom Score-31; eGFR, estimated glomerular filtration rate; HbA1c, glycated hemoglobin; IQR, interquartile range; KCCQ, Kansas City Cardiomyopathy Questionnaire; NAC, National Amyloidosis Centre stage; NT-proBNP, N-terminal prohormone of brain natriuretic peptide; NYHA, New York Heart Association stage; SD, standard deviation. Categorical data are displayed as counts (percentage). Metric data are given as mean ± standard deviation in cases with a normal distribution and as median (interquartile range) in cases with a non-normal distribution, which is assessed utilizing the Shapiro–Wilk test. *p*-Values for metric variables are derived from the t-test for independent samples and the Mann–Whitney U test, respectively. For binary/categorical variables, the chi-square test is utilized.

**Table 3:** Correlation of the Composite Autonomic Symptom Score-31 and Kansas City Cardiomyopathy Questionnaire.

Variable		Orthostatic Intolerance Score	Vasomotor Score	Secretomotor Score	Gastrointestinal Score	Bladder Dysfunction Score	Pupillomotor Dysfunction	Overall Score
**Vasomotor score**	Correlation	*0.12*						
	*p*-value	*0.527*						
**Secretomotor score**	Correlation	*0.45*	*0.24*					
	*p*-value	***<0.001***	*0.072*					
**Gastrointestinal score**	Correlation	*0.32*	*0.30*	*0.39*				
	*p*-value	***0.005***	***0.009***	***<0.001***				
**Bladder dysfunction score**	Correlation	*0.15*	*0.31*	*0.39*	*0.41*			
	*p*-value	*0.438*	***0.007***	***<0.001***	***<0.001***			
**Pupillomotor score**	Correlation	*0.35*	*0.15*	*0.37*	*0.31*	*0.29*		
	*p*-value	***0.002***	*0.438*	***0.001***	***0.008***	***0.013***		
**Overall Score**	Correlation	*0.84*	*0.31*	*0.71*	*0.65*	*0.44*	*0.50*	
	*p*-value	***<0.001***	***0.007***	***<0.001***	***<0.001***	***<0.001***	***<0.001***	
**KCCQ physical limitations score**	Correlation	*−0.27*	*−0.29*	*−0.32*	*−0.30*	*−0.33*	*−0.09*	*−0.39*
	*p*-value	***0.029***	***0.016***	***0.006***	***0.011***	***0.006***	*0.635*	***<0.001***
**KCCQ symptom frequency score**	Correlation	*−0.32*	*−0.30*	*−0.48*	*−0.46*	*−0.44*	*−0.21*	*−0.54*
	*p*-value	***0.006***	***0.009***	***<0.001***	***<0.001***	***<0.001***	*0.145*	***<0.001***
**KCCQ quality of life score**	Correlation	*−0.34*	*−0.27*	*−0.39*	*−0.44*	*−0.36*	*−0.18*	*−0.51*
	*p*-value	***0.004***	***0.029***	***<0.001***	***<0.001***	***0.002***	*0.305*	***<0.001***
**KCCQ social limitations score**	Correlation	*−0.35*	*−0.23*	*−0.37*	*−0.36*	*−0.35*	*−0.08*	*−0.48*
	*p*-value	***0.004***	*0.137*	***0.002***	***0.003***	***0.005***	*0.635*	***<0.001***
**KCCQ-12** **Overall score**	Correlation	*−0.37*	*−0.31*	*−0.44*	*−0.44*	*−0.42*	*−0.17*	*−0.55*
	*p*-value	***0.001***	***0.008***	***<0.001***	***<0.001***	***<0.001***	*0.322*	***<0.001***

COMPASS-31, Composite Autonomic Symptom Score-31; KCCQ-12, Kansas City Cardiomyopathy Questionnaire.

**Table 4:** Linear regression analysis demonstrating the association between patient characteristics and the COMPASS-31 score.

Variable	Coef.	95% CI	*p*-Value	Coef.	95% CI	*p*-Value
	Crude			Multivariate Adjustment		
Age	0.038	−0.394–0.469	*0.864*			
Female sex	4.489	−2.767–11.746	*0.223*			
ATTRv	3.786	−6.293–13.866	*0.459*			
NYHA	6.712	3.282–10.142	***<0.001***	0.316	−3.507–4.139	*0.870*
NAC stage	3.292	−0.039–6.623	*0.053*			
Diabetes mellitus	−2.353	8.964–4.258	*0.483*			
Disease-modifying therapy ≥ 6 months	−3.733	−9.225–1.759	*0.181*			
KCCQ	−0.360	−0.461–−0.260	***<0.001***	−0.356	−0.481–−0.227	***<0.001***

ATTRv, variant amyloid transthyretin; CI, confidence interval; NAC, National Amyloidosis Centre stage; NYHA, New York Heart Association stage; KCCQ, Kansas City Cardiomyopathy Questionnaire.

## Data Availability

The data underlying this article will be shared on reasonable request to the corresponding author.

## Abbreviations

The following abbreviations are used in this manuscript:

ATTR	amyloid transthyretin
ATTR-CM	amyloid transthyretin cardiomyopathy
CA	cardiac amyloidosis
COMPASS-31	Composite Autonomic Symptom Score-31
eGFR	estimated glomerular filtration rate
HbA1c	glycated hemoglobin
HF	heart failure
IQR	interquartile range
KCCQ	Kansas City Cardiomyopathy Questionnaire
NAC	National Amyloidosis Centre
NYHA	New York Heart Association functional class
NT-proBNP	N-terminal prohormone of brain natriuretic peptide
